# Dependency of Calcium Alternans on Ryanodine Receptor Refractoriness

**DOI:** 10.1371/journal.pone.0055042

**Published:** 2013-02-04

**Authors:** Enric Alvarez-Lacalle, Inma R. Cantalapiedra, Angelina Peñaranda, Juan Cinca, Leif Hove-Madsen, Blas Echebarria

**Affiliations:** 1 Departament de Física Aplicada, Universitat Politècnica de Catalunya-Barcelona Tech, Barcelona, Spain; 2 Cardiology Service IIB-Sant Pau, Hospital de la Santa Creu i Sant Pau, Barcelona, Spain; 3 Cardiovascular Research Centre CSIC-ICCC, Hospital de la Santa Creu i Sant Pau, Barcelona, Spain; Baylor College of Medicine, United States of America

## Abstract

**Background:**

Rapid pacing rates induce alternations in the cytosolic calcium concentration caused by fluctuations in calcium released from the sarcoplasmic reticulum (SR). However, the relationship between calcium alternans and refractoriness of the SR calcium release channel (RyR2) remains elusive.

**Methodology/Principal Findings:**

To investigate how ryanodine receptor (RyR2) refractoriness modulates calcium handling on a beat-to-beat basis using a numerical rabbit cardiomyocyte model. We used a mathematical rabbit cardiomyocyte model to study the beat-to-beat calcium response as a function of RyR2 activation and inactivation. Bi-dimensional maps were constructed depicting the beat-to-beat response. When alternans was observed, a novel numerical clamping protocol was used to determine whether alternans was caused by oscillations in SR calcium loading or by RyR2 refractoriness. Using this protocol, we identified regions of RyR2 gating parameters where SR calcium loading or RyR2 refractoriness underlie the induction of calcium alternans, and we found that at the onset of alternans both mechanisms contribute. At low inactivation rates of the RyR2, calcium alternans was caused by alternation in SR calcium loading, while at low activation rates it was caused by alternation in the level of available RyR2s.

**Conclusions/Significance:**

We have mapped cardiomyocyte beat-to-beat responses as a function of RyR2 activation and inactivation, identifying domains where SR calcium load or RyR2 refractoriness underlie the induction of calcium alternans. A corollary of this work is that RyR2 refractoriness due to slow recovery from inactivation can be the cause of calcium alternans even when alternation in SR calcium load is present.

## Introduction

Despite the important role of electro-mechanical alternans in cardiac arrhythmogenesis [1], [2], its molecular origin is not well understood. This phenomenon has been associated with alternation in both ionic currents and in the cytosolic calcium transient. The latter has been linked to a dysfunction of sarcoplasmic reticulum (SR) calcium uptake [3], [4], or release [4], [5], [6], [7]. Indeed, several reports [5], [7] seem to support the hypothesis that the origin of alternans could lie in a steep relationship between SR calcium load and calcium release [4]. This steep relation has been explained as a dependence of the operating state of the ryanodine receptor (RyR2) with the SR calcium bound to calsequestrin [8], thus implying a stronger release at high calcium loads.

Nevertheless, cytosolic calcium alternans has been observed both in the absence and presence of concurrent fluctuations in SR calcium loading [9], [10], [11]. Recently, Shkryl et al [11] have confirmed the presence of alternans without SR calcium fluctuations and related it to incomplete recovery in refractoriness of SR calcium release. This suggests that, besides calcium loading, other properties of the SR, such as activation of the ryanodine receptor (RyR2) [5], [6], inactivation of the RyR2 [12], [13], recovery of the RyR2 from inactivation [12], [14], and termination of calcium release through the RyR2 [15], [16], may all intervene in the regulation of the beat-to-beat stability of the cytosolic calcium transient.

To address this issue, a major challenge lies in the difficulty of using experimental animal or cell models to resolve the specific contribution of a single property of the SR to the calcium transient and its beat-to-beat stability. Most often, manipulation of one parameter affects the state of several others, thus hampering quantification of its specific contribution. We here attempt to circumvent this problem by developing a novel numerical protocol applied to a computer model of a rabbit ventricular myocyte, where we can specifically change the dynamics of SR loading and RyR2 gating, and investigate the mechanisms responsible for the induction of calcium alternans, under different operating conditions of the RyR2.

## Methods

We used a description of a rabbit ventricular myocyte based on the model described by Shannon et al [17]. The same formal equations were used, but differences in the values of some parameters of the calcium dynamics were introduced. The description of the RyR2 considers transitions among four states, one open, one closed, and two inactivated. The nomenclature and associated reaction equations for the RyR2 are shown in Figure S1 of Appendix S1. Activation and inactivation rates, given by the constants *k_a_*, and *k_i_*, were systematically changed in order to analyze their effect on the beat-to-beat response (see Table S1 in Appendix S1 for a summary of changes in the parameters). We have measured the alternans amplitude, defined as the difference in peak cytosolic calcium at two consecutive beats, as a function of activation and inactivation rates, and for different values of the pacing period and the RyR2 recovery time from inactivation. The myocyte model by Shannon et al [17] using the original parameters for *k_a_*, and *k_i_* does not give rise to calcium alternans neither at normal pacing rates (3 Hz) nor when the pacing interval is shortened further. However, we find that changing activation and inactivation rates can produce the appearance of long (of at least 20 beats) transient calcium alternans at 5 Hz (Figure S2 in Appendix S1), as observed in isolated rabbit cardiomyocytes [18]. Larger changes in activation and inactivation rates generate alternans even at normal pacing rates (∼3 Hz). We also considered several values of the time for RyR2 recovery from inactivation, τ_r_, τ_r_ = 200 ms (the original value), 750 ms, and 1500 ms. In most simulations we used as benchmark value τ_r_ = 750 ms. This value agrees with the refractoriness of calcium release measured in other reports [9], [15], [19]. We checked that this benchmark value is consistent with experiments on the restitution of the calcium transient [9] (see Appendix S1).

We developed a novel numerical protocol to analyze the specific effects on calcium handling dynamics of RyR2 activation, inactivation and recovery from inactivation, as well as SR calcium loading. In this protocol, the myocyte was stimulated at a constant pacing rate until steady state was reached. Then, we compared this steady state response with simulations where alternations in SR Ca load, or alternations in the recovery level of RyR2s were eliminated. The key aspect of this scheme is the procedure to eliminate alternations in SR Ca load or in the level of recovered RyR2s, which we achieved using a dynamic clamping protocol. The specific details are described in the next subsection together with a discussion on how they fit in the general problem of uncovering the mechanism behind the presence of calcium alternans.

### Dynamic Clamping Protocols

During alternans, the intracellular cytosolic calcium transient alternates from beat to beat. Whenever this happens there is a corresponding alternation in both the pre-systolic SR calcium load and the level of RyR2 ready to open (not inactivated, i.e. in state R of Figure S1 in Appendix S1). These two types of oscillations are directly related with two mechanisms proposed to account for calcium alternans in the literature. One states that a change in the calcium loading process leads to cytosolic calcium alternans (calcium alternans due to SR calcium load). The other states that the level of RyR2s recovered from inactivation oscillates. An ideal experiment to discern the underlying mechanism would require eliminating the alternation in one of the variables and test if the cytosolic calcium alternans persists. If it does not persist, we can be confident that alternation in this variable is a necessary condition for cytosolic calcium alternans. To be sure that there are no spurious effects this modification must be done without changing the release process, and with minimal changes in the overall calcium dynamics.

While eliminating the oscillation without affecting release is unfeasible in the laboratory, the protocol we developed allows us to implement it in the mathematical myocyte model via a dynamic clamping of the variables involved. We describe first the details of the dynamic clamping of the SR calcium load and then of the level of recovered RyR2s. Both clamping protocols can be activated separately or simultaneously. In the latter case, cytosolic calcium alternans should disappear if there is no other intervening mechanism.

### Clamping of the SR Ca Load

**Figure 1:**
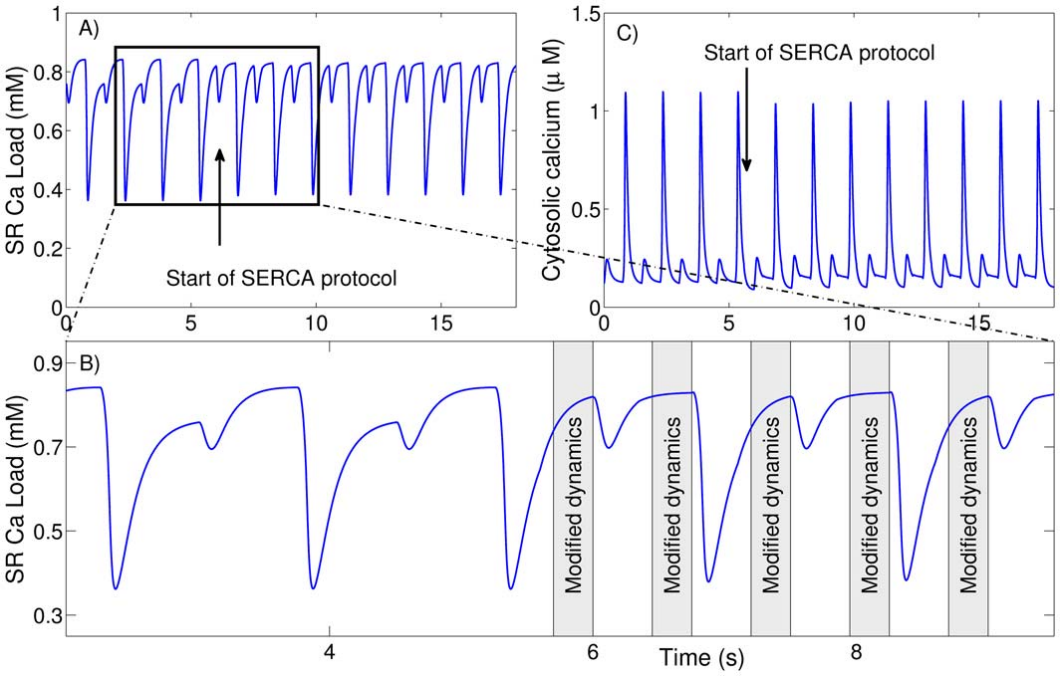
Dynamic protocol for eliminating oscillations in pre-systolic SR Ca load. Panel A) indicates the moment where the protocol is activated while panel B) shows the intervals where the SERCA dynamics are modified to pump more strongly in order to make the level of SR Ca load reach the same level on each beat. In this case, this level corresponds to 0.83 mM, which is the maximum SR Ca load obtained before the activation of the clamping-protocol. Panel C) shows that, in this case, calcium alternans persists even when oscillations in pre-systolic SR Ca load are eliminated.

Figure 1 illustrates the workings of this protocol. Initially, the cell is paced at a given rate using our standard numerical model until calcium alternans has stabilized (see Figure 1A). Then, starting at any given beat, we change the dynamics of the sarco/endoplasmic reticulum Ca2+-ATPase (SERCA) uptake during the last 150 ms of diastole so that the SR calcium load achieves the same value 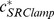 before each external excitation (see Figure 1B). The value of 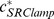 is taken to be equal to the maximum pre-systolic SR calcium load during the non-clamped dynamics. During the external excitation and SR calcium release, the original SERCA current is used. In this way we do not affect the dynamics of calcium release and re-uptake. More importantly, the dynamics is only affected when all the variables are close to their equilibrium values. As seen in Figure 1B–C the result is a clamped dynamics where the SR Ca load before each calcium release is constant and where the evolution of the cytosolic calcium transient (Figure 1C) indicates if calcium alternans is affected or not by this clamping.

The procedure can be summarized as testing whether calcium alternans disappears when the SERCA current is set as follows:
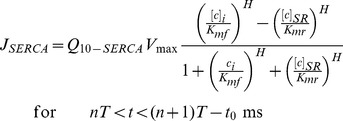
(1)

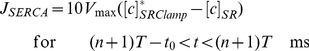
(2)where typically *t*_0_ = 150 ms (we use *t*_0_ = 75–100 ms for *T*<240 ms). Equation (1) is the original SERCA uptake, with the parameters given in [17] active during the external excitation, calcium release and first stages of the reuptake. During the last *t*_0_ before each beat we substitute the SERCA uptake for a stronger current, which keeps pumping calcium from the cytosol until 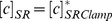.

### Clamping of RyR2 Recovery

**Figure 2:**
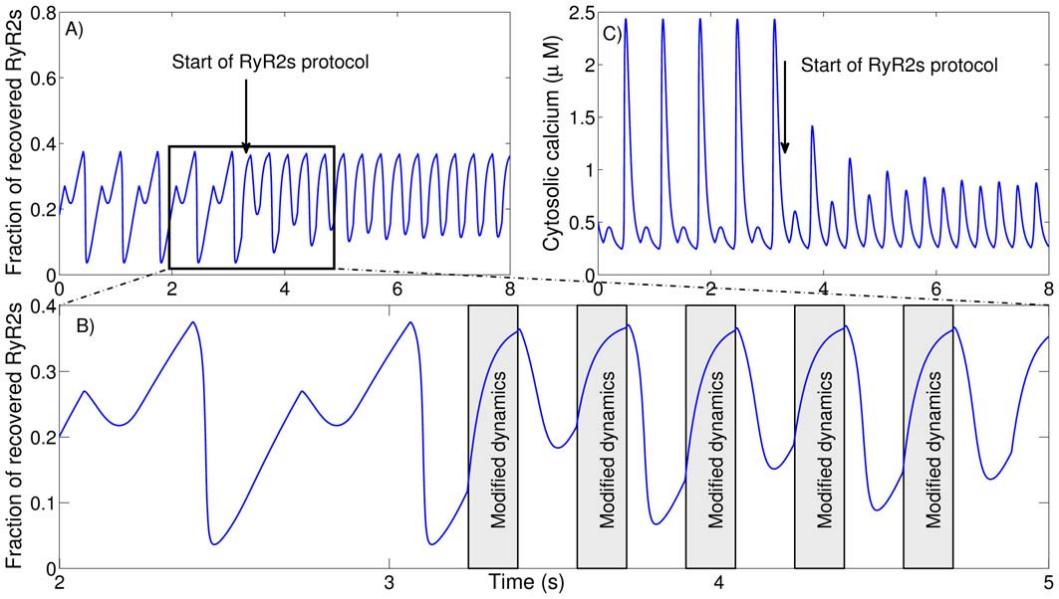
Dynamic protocol for eliminating oscillations in the pre-systolic level of recovered RyRs. Panel A) indicates the moment where the protocol is activated while panel B) shows the intervals where the recovery dynamics of RyR2s is accelerated at the same time that only a fraction of them remain active. This fraction corresponds to a recovery of 37% of the total RyR2. This is the maximum level present before the clamping-protocol is started, and it is the one we aim to reach at the end of diastole. Panel C) shows that, in this case, calcium alternans is eliminated when oscillations in the level of recovered RyR2s are eliminated.

Following the same idea of the previous clamping protocol, the clamping of RyR2 recovery is achieved changing its dynamics during the 150 ms before each calcium release (see Figure 2). These changes in the RyR2 are applied to eliminate dynamically the oscillation in the pre-systolic ratio of recovered RyR2 (*R* state) without affecting calcium release and uptake. In this clamping protocol a number of RyR2 channels are disabled at a time 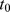 before the following beat, with an acceleration of the recovery rate of the remaining RyR2s, so that the final recovery level of receptors before the calcium release is set to a given value *R_clamp_*. The equations for the total number of states read:
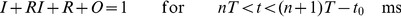
(3)

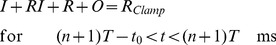
(4)with the recovery rate changing form the original value to τ_r_ = 50 ms (*k*_im_ = 0.02 ms^−1^) in the last *t*_0_ before each external excitation. To make both the unclamped and clamped dynamics equivalent, *R_clamp_* is taken to be the maximum number of pre-systolic non-inactivated channels obtained in the presence of alternans when no clamping protocol is used (see Figure 2B). In effect, this means disabling a ratio of 1- *R_clamp_* at time (*n*+1)*T*-*t*_0_ and leaving *R_clamp_* active as indicated in Eq. (4).

## Results

### Effect of RyR2 Activation and Inactivation on the Induction of Alternans

To validate the model, we first verified that changes in RyR2 activation and inactivation rates could produce alternans at fast pacing rates. Additionally, Picht et al [9] observed that calcium release increases with rest time, even if the content of the SR decreases (and the I_CaL_ current has fully recovered), and they suggested that this post-rest potentiation is due to a slow recovery from refractoriness of RyR2 calcium release. In the current model, refractoriness is given by the recovery of the RyR2 from inactivation. We find that, for a recovery time of τ_r_ = 750 ms, the model reproduces qualitatively the post-rest potentiation of RyR2 calcium release, as shown in Figure S6 in Appendix S1.

**Figure 3:**
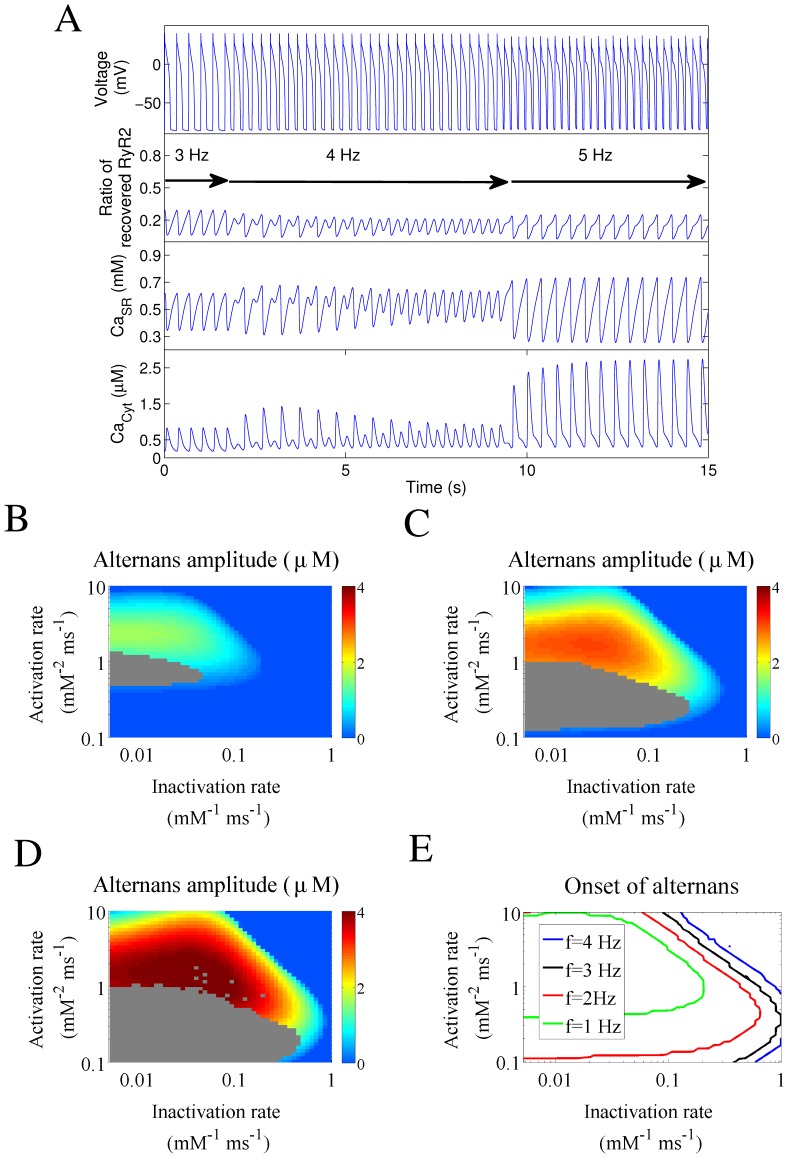
Slowing of RyR2 activation or inactivation induces calcium alternans at physiological pacing rates. A) The effect of increasing the stimulation frequency from 3 Hz to 5 Hz on trasmembrane potential (top panel), fraction of recovered RyRs (top middle panel), SR calcium load (lower middle panel) and cytosolic calcium (lower panel) for fixed activation and inactivation rates of *k_a_* = 8.5 mM^−2^ ms^−1^, *k_i_* = 0.17 mM^−1^ ms^−1^ with a recovery time from inactivation of τ_r_ = 1/*k_im_* = 750 ms. B), C), and D) Color-code graphs showing the amplitude of alternations in the calcium transient amplitude as a function of RyR2 activation and inactivation at a pacing rate of 1 Hz (B), 2 Hz (C), and 3 Hz (D). The horizontal axis represents the RyR2 inactivation rate, while the vertical axis represents the RyR2 activation rate. The alternans amplitude, defined as the difference in peak cytosolic calcium between two consecutive beats, is given in color code with blue representing no alternans and dark red corresponding to strong alternations in peak values. The gray area represents cases where a complex beat-to-beat behavior is observed, including 3∶1 or 4∶1 rhythms, or seemingly chaotic dynamics. E) Borders for the transition to cytosolic calcium alternans obtained with different pacing frequencies.

The original parameters in the Shannon model did not present calcium alternans at any frequency. However, Figure 3A shows that reduced activation and inactivation (*k_a_* = 8.5 mM^−2^ ms^−1^, *k_i_* = 0.17 mM^−1^ ms^−1^, or 85% and 35% of the original values), lead to calcium alternans. Alternans first appeared transiently when the pacing rate was increased from 3 Hz to 4 Hz, and thereafter became sustained at 5 Hz. Notice that changes in the RyR2 produced oscillations in the SR calcium loading despite the fact that neither changes in the loading properties of the SERCA pump, nor in the calsequestrin (CSQN) levels of the SR were introduced. Alternans was not only associated with oscillations in the SR calcium loading (*c_SR_*), but also with alternations in the level of recovered RyR2s ready to open on each stimulation. Subsequently, the model was used to examine how changes in the RyR2 activation-inactivation rates were able to induce alternans even at normal pacing rates (3 Hz). Figure 3 shows that cytosolic calcium alternans appears when either activation or inactivation rates are diminished. The onset of alternans appeared at different combinations of activation and inactivation rates, defining a boundary between uniform and alternating responses (Figures 3B, C, D), which moved depending on stimulation frequency (Figure 3E). As expected, the area of alternating responses increased as the stimulation frequency was increased (Figure 3E). For some parameters (gray area in Figures 3B, C and D) we also observed the presence of a complex beat-to-beat behavior, including 3∶1 or 4∶1 rhythms, or seemingly chaotic dynamics.

To check that the observed alternations were due to instability in the calcium handling dynamics, with no significant effect of voltage dynamics on their generation, we repeated the previous simulations using an AP clamp protocol, obtaining the same results (Figure S3 in Appendix S1). As we proceed to show, cytosolic calcium alternans appeared due to oscillations in either SR calcium loading or RyR2 dynamics.

### Mechanisms Underlying Cytosolic Calcium Alternans

In order to investigate how SR calcium load and fractional recovery of the RyR2s from inactivation contributed to cytosolic calcium alternans, we clamped either of these variables and determined which of the clamping procedures was able to eliminate the cytosolic calcium alternation. The simultaneous clamping of the SR Ca load and of the rate of recovered RyR2 always eliminated alternans, both with current and AP clamp. Thus, in all the cases discussed here the mechanism for calcium alternans is related to either SR Ca load, recovery of the RyR2 from inactivation, or both.

**Figure 4:**
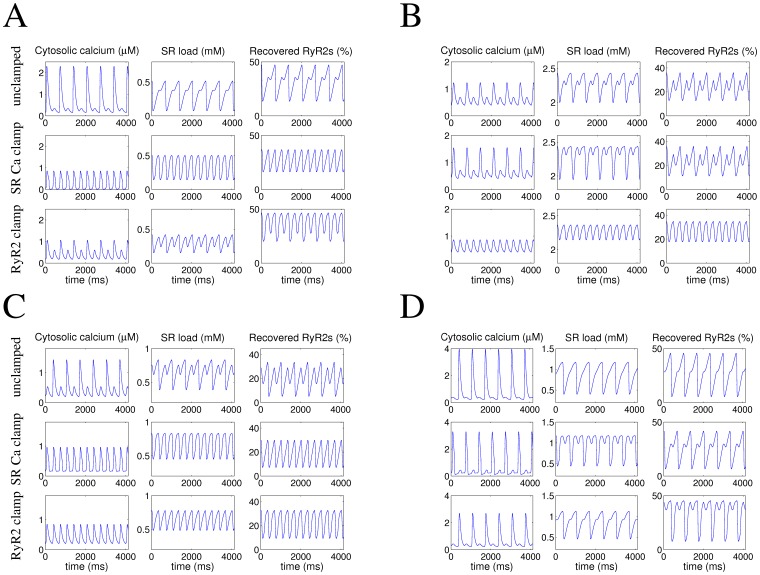
Contribution of SR calcium load and recovery of RyR2 from inactivation to the induction of calcium alternans.

Figure 4A shows an example where only a clamping of the SR calcium load eliminated alternans, demonstrating that, in this case, alternation in SR calcium load is necessary for the induction of alternans. Figure 4B shows an example where calcium alternans disappears only when the fraction of recovered RyR2s is clamped, and thus the responsible mechanism is alternation in the number of RyR2 that are recovered from inactivation. Figures 4C and 4D show examples where clamping of either variable eliminates calcium alternans or neither of them alone does. Thus, in Figure 4C both mechanisms are necessary to sustain alternans, while in Figure 4D either of them by itself is able to maintain it, without being necessary the presence of the other.

**Figure 5:**
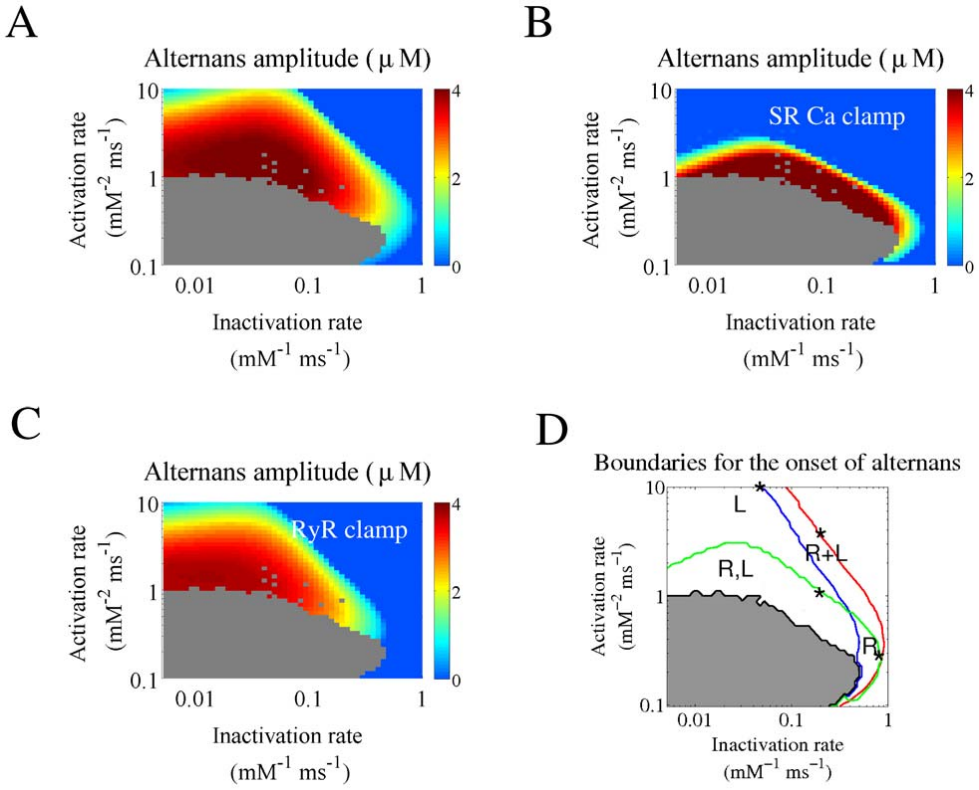
Mechanism underlying the onset of alternans at different activation and inactivation rates. A) Color-code graph showing the amplitude of the cytosolic alternans at 3 Hz. Blue indicates the absence of alternans and dark red the biggest alternation. B) The same simulations as in A) but with SR Ca loading clamped at presystolic values. C) The same simulations as in A) but with the fraction of recovered RyRs clamped at presystolic values. D) Lines denoting the onset of alternans under: normal (un-clamped) conditions (red line), clamped SR Ca load (green line), and clamped fraction of recovered RyRs (blue line). The gray area represents the region with irregular behavior under un-clamped conditions. These lines delimitate the regions where alternations in SR calcium load (“L”) and RyR2 recovery (“R”) are responsible for calcium alternans. “R,L” indicate the region where alternations in either recovery of RyR2s from inactivation or SR Ca load are capable of maintaining alternans, while “R+L” requires alternation in both mechanisms to sustain cytosolic calcium alternans. The four asterisks correspond to the four examples shown in Figure 4.

Each of these examples was obtained with different combinations of activation and inactivation rates. To determine which mechanisms can sustain calcium alternans for any given combination of the RyR2 activation and inactivation rates, we repeated the simulations shown in Figure 3D clamping either SR calcium load (Figure 5B) or the fraction of recovered RyR2s (Figure 5C). When the SR calcium load was clamped (Figure 5B), the boundary denoting the onset of alternans moved to lower values of activation or inactivation, but there was still a large area where alternans is present. This indicated that recovery of the RyR2 from inactivation was able to sustain alternans in that region. On the other hand, when the fraction of recovered RyR2s was clamped (Figure 5C), calcium alternans was also maintained in a large area. Therefore, combining Figures 5A, B, and C allowed us to identify the regions where (see Table 1): 1) alternation in SR calcium load is the only mechanism underlying calcium alternans (region “L”); 2) recovery of the RyR2 from inactivation is the responsible mechanism (region “R”); 3) both mechanisms are necessary (region “R+L”); 4) either mechanism is able to sustain alternans (region “R, L”). Figure 5D shows how these four regions are distributed as a function of activation and inactivation rates for a pacing frequency of 3 Hz.

**Table 1:** Mechanisms of calcium alternans.

		Mechanism			
		“R”	“L”	“R+L”	“R, L”
Clamping protocol	SR Clamping	Alternans Persists	Alternans Disappears	Alternans Disappears	Alternans Persists
	RyR2 Clamping	Alternans Disappears	Alternans Persists	Alternans Disappears	Alternans Persists

“R” stands for alternans due to alternation in RyR2 recovery from inactivation, “L” stands for alternans due to alternation in SR Ca load, “R+L” stands for alternans that require both oscillations in the recovery of RyR2s and in SR Ca load. Finally, “R,L” stands for alternans where both mechanisms contribute but either can sustain it. The case where both protocols were applied at the same time is not shown since, in all cases, alternans disappeared.

To further understand the presence of alternans when SR load does not alternate, we considered an idealized situation where: 1) stimulation was done using an action potential clamp, and 2) the SR calcium and 3) the subsarcolemmal calcium were fixed at a constant concentration at all times. This ensures that, if alternans still appears, the RyR2 dynamics is its only possible source. From a mathematical analysis of this case (see Section 2 in Appendix S1) we demonstrate the presence of an instability that gives rise to alternans, through a period-doubling bifurcation (Figure S4 in Appendix S1). The instability is inherent to the RyR2 dynamics and requires a stimulation period shorter than its recovery time from inactivation (Figure S5 in Appendix S1).

**Figure 6:**
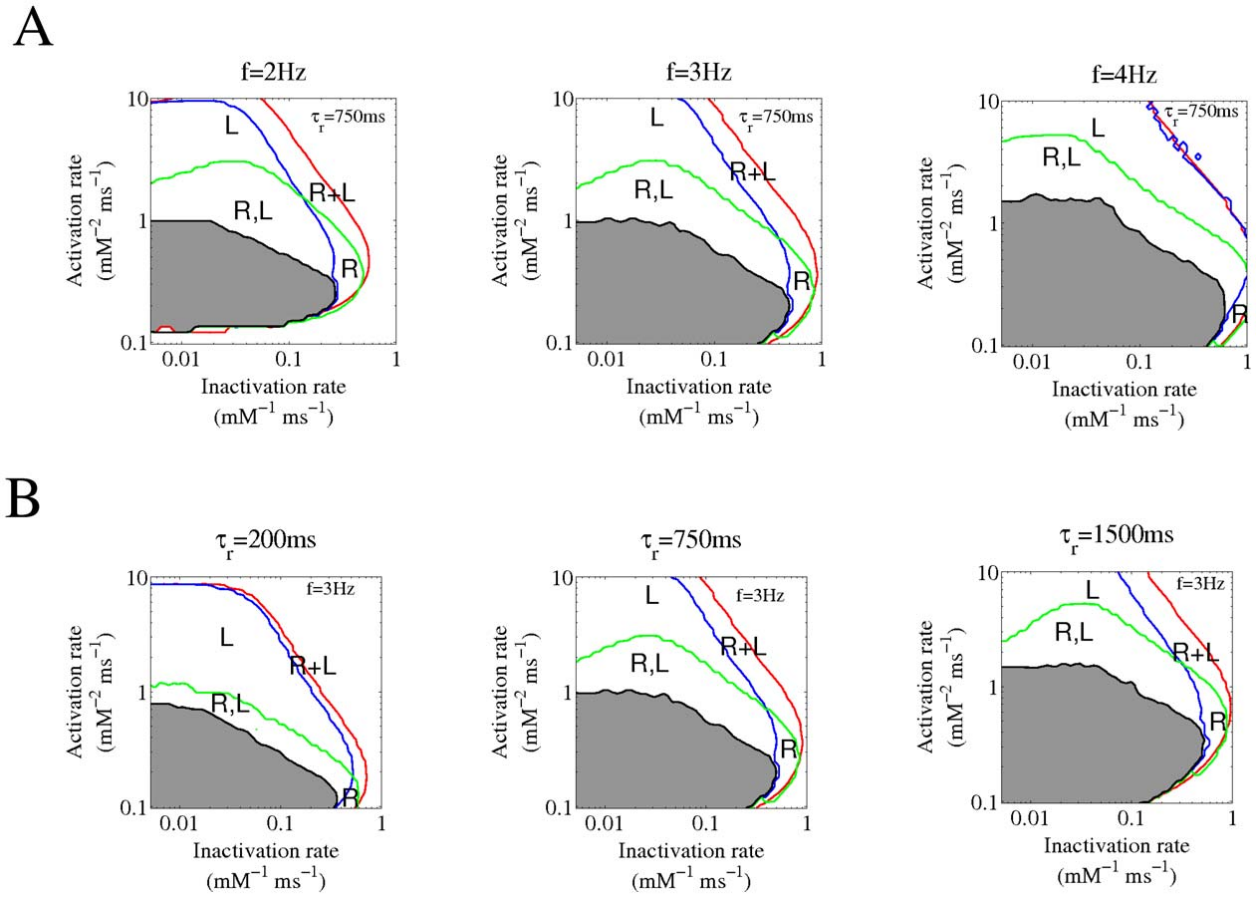
Mechanisms responsible for the onset of alternans for different pacing rates and RyR2 recovery times from inactivation. A) The limits for the onset of alternans are shown as in Figure 5D (reproduced here in the central panels), for different pacing frequencies: 2 Hz, 3 Hz, 4 Hz (with τ_r_ = 750 ms). B) The limits for the onset of alternans for different values of the RyR2 recovery time τ_r_: 200 ms, 750 ms, 1500 ms (at a pacing frequency of 3 Hz).

We then investigated how the stimulation frequency affects the relative relevance of the different mechanisms, recalculating Figure 5D at different pacing rates (2 Hz, 3 Hz and 4 Hz) and the results are summarized in Figure 6A.

### Effect of Changes in the Recovery Time of the RyR2 from Inactivation

**Figure 7:**
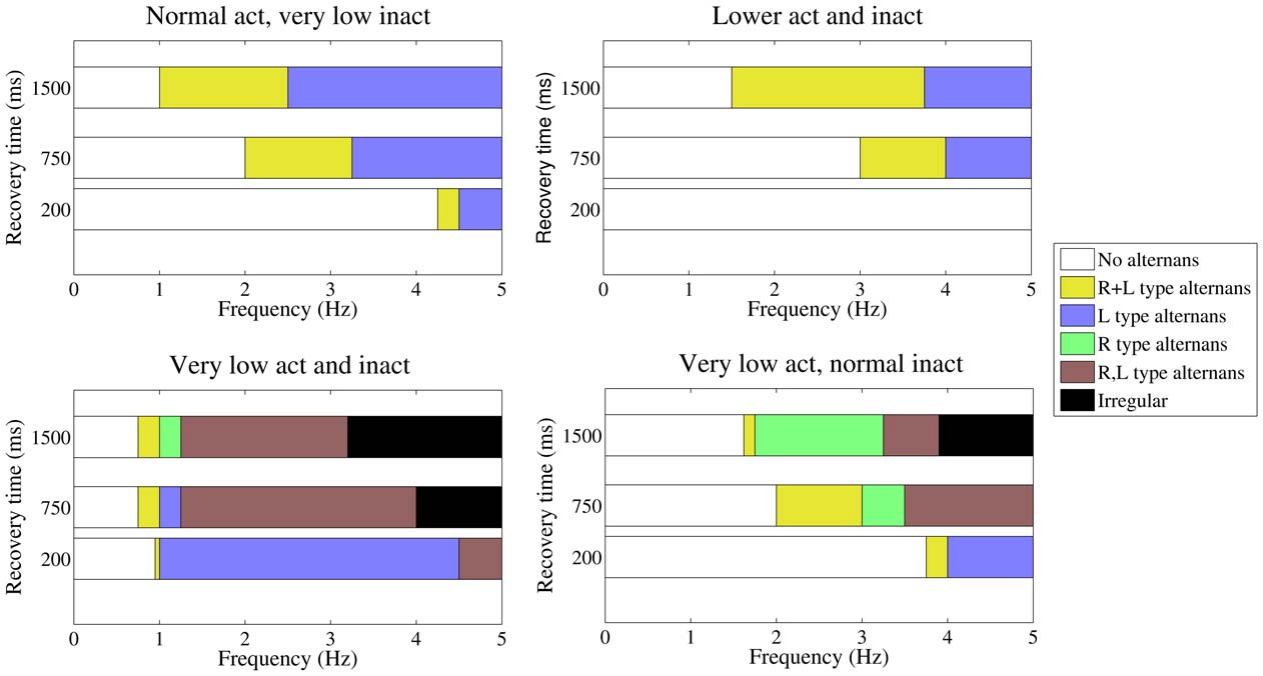
Mechanism underlying the onset of alternans at different pacing frequencies and RyR2 recovery times . The four panels illustrate how the mechanism underlying the induction of cytosolic calcium alternans varies with the stimulation frequency and RyR2 recovery from inactivation. Each panel has three rows of color bars, which indicates the responsible mechanism for the induction of alternans at the different stimulation frequencies. The top bar represents slow RyR2 recovery (τ_r = _1500 ms), the middle bar intermediate RyR2 recovery (τ_r_ = 750 ms) and the lower bar fast RyR2 recovery from inactivation (τ_r_ = 200 ms). Colors green, purple, yellow, and brown correspond, respectively, to the regimes R, L, R+L, and R,L of Table 1. Black indicates frequencies where irregular behavior is present. The parameters for activation and inactivation are: top panels, left: *k*_a_ = 10 mM^−2^ ms^−1^, *k*_i_ = 0.05 mM^−1^ ms^−1^, right: *k*_a_ = 3.5 mM^−2^ ms^−1^, *k*_i_ = 0.2 mM^−1^ ms^−1^; lower panels, left: *k*_a_ = 1.0 mM^−2^ ms^−1^, *k*_i_ = 0.1 mM^−1^ ms^−1^, right: *k*_a_ = 0.6 mM^−2^ ms^−1^, *k*_i_ = 0.5 mM^−1^ ms^−1^.

Figure 6B shows that the boundaries of calcium alternans enlarge as the time for recovery of the RyR2 from inactivation increases from 200 ms to our standard value of 750 ms, and further to 1500 ms. To expand this analysis to different frequencies, four representative sets of values for the activation and inactivation rates were selected, corresponding to regions “R”, “L”, “R, L”, and “R+L”. Figure 7 shows how the stimulation frequency and the recovery time affect the appearance of alternation. Notice that for the four sets of parameters considered, increasing the stimulation rate, the onset of alternans occurred first (i.e. at the lowest stimulation frequency) under conditions where both mechanisms are required (“R+L”). Similarly, diminishing the RyR2 recovery time reduced the band of frequencies where recovery from inactivation contributed to the maintenance of calcium alternans. Finally, the contribution of SR calcium load to the maintenance of calcium alternans became more predominant at high frequencies.

## Discussion

### Main Findings

The present study has used a mathematical myocyte model and a numerical clamping protocol to map beat-to-beat changes in the cytosolic calcium transient as a function of RyR2 activation and inactivation as well as the identification of domains where SR calcium load and/or RyR2 recovery from inactivation contribute to the induction of calcium alternans. This approach makes it possible to identify transition zones where one predominant mechanism is substituted by another, and a characterization of how the transition zones depend on the stimulation frequency, SR calcium load and the RyR2 recovery time. This model represents a novel tool to predict how mutations or drugs that affect RyR2 gating properties will modify the beat-to-beat stability of calcium handling. Importantly, this model also demonstrates that even when experimental data shows concurrent alternations in calcium load and the cytosolic calcium transient, this does not necessarily imply that alternation in calcium load is the underlying mechanism.

### Validation and Limitations of the Model

The current approach used a validated rabbit ventricular myocyte model [17] that incorporates realistic features of intracellular calcium handling, and it faithfully reproduced calcium dynamics under steady-state conditions. Taking into account that elevation of the stimulation frequency induces beat-to-beat alternations in the cytosolic calcium transient upon elevation of the stimulation frequency [4], [7], [9], we identified modifications of the gating properties of the RyR2 (such as its activation time, inactivation time, and/or the recovery time from inactivation) that were necessary for the model to reproduce these phenomena.

For the present analysis activation times ranged from 0.01 to 1x the values used by Shannon et al [17]. These values were chosen to cover the range where alternans could be induced, but it has also been shown that mutations in the TM10 region of the RyR2 can in fact reduce the RyR2 sensitivity to Ca^2+^ activation by as much as 1,000 fold less than the wild type [20]. For inactivation times, we used values that ranged from 0.01 to 2x the value employed by Shannon et al [17], which is also consistent with reports showing that the failure to completely terminate Ca^2+^ release following channel stimulation may arise as a consequence of a loss of Ca^2+^-dependent inactivation (8- to 10-fold) [21]. The present RyR2 model includes inactivation processes on a fast time scale, giving rise to refractoriness in release. This is contrary to the behavior in single RyR2 dynamics, where refractoriness of gating has never been observed and inactivation processes are too slow to be significant on a beat-to-beat time scale. Thus, the RyR2 dynamics in the present model must be understood as phenomenologically modeling the collective behavior of several RyR2s or clusters of RyRs that generate calcium sparks, where long time refractoriness has been observed.

The model used does not consider stochastic variations among calcium release units (CaRUs), and can therefore not account for asynchronous release. Indeed, the model does not present calcium waves, although complex or chaotic beat-to-beat patterns are observed (gray areas in Figures 3 and 5). However, this does not represent a serious limitation when analyzing synchronized responses where calcium waves are not present.

Besides the specific ventricular myocyte model used here, the protocol employed to uncover the mechanism behind alternans is of general use and can be applied to any other whole cell cardiomyocyte model.

### Effect of RyR2 Activation and Inactivation on the Induction of Calcium Alternans

The two-dimensional mapping of the beat-to-beat response as a function of RyR2 activation and inactivation showed that lowering either RyR2 activation or RyR2 inactivation leads to the induction of calcium alternans. Moreover, it showed that elevation of the stimulation frequency moves the boundary for the induction of calcium alternans towards faster activation and/or inactivation rates. In this context, low levels of RyR2 activation have previously been related to the onset of alternans in experiments where the RyR2 open probability (P_o_) was decreased with tetracaine or intracellular acidification, which have been shown to produce variability in the Ca^2+^ transient [6]. Considering that decreased RyR2 open probability may arise from either slower RyR2 activation or faster RyR2 inactivation, the present model confirms that slower RyR2 activation does indeed promote calcium alternans but shows that faster RyR2 inactivation prevents the induction of calcium alternans, suggesting that tetracaine and intracellular acidification likely decreases the RyR2 open probability by slowing its activation.

### Role of SR Calcium Load and RyR2 Recovery from Inactivation on the Induction of Calcium Alternans

Our results show that slowing of inactivation leads to calcium alternans, which is abolished when SR calcium loading is clamped. This indicates that SR Ca load oscillations are necessary for alternans in this case. Indeed, alternation in SR Ca load is a widely accepted explanation for cytosolic calcium alternans, which relies on a steep relationship between SR calcium loading and Ca^2+^ release that makes any small difference in loading in alternating beats prone to grow, through a feedback mechanism (see Shiferaw et al [4]). Physiologically, the origin of this steep relationship could be an effect of luminal calcium on the RyR2 open probability [12], [22] or it could be caused by desynchronized calcium release in the form of calcium waves, that mark an abrupt change in the slope of the SR calcium load-release relationship [23]. Here, we reproduce, by changing RyR2 refractoriness, alternations in cytosolic calcium transient that are necessarily linked to sarcoplasmic reticulum calcium fluctuations. Very low inactivation rates correspond, effectively, to situations where the inactivated state is irrelevant since the rate of RyR2 which transit to inactivation is very low. This leads to an effective two-state model of RyR2, which presents alternation due to the steep relationship between SR load and release. Alternans due to SR Ca load has also been obtained numerically by Restrepo et al [8] using different dynamics of the RyR2, with two closed and two open states.

Calcium alternans is also induced by a slowing of RyR2 activation, if inactivation is non-negligible. In this case, alternans is abolished by clamping RyR2 recovery but not by clamping SR Ca load, indicating that incomplete RyR2 recovery is the underlying mechanism. The physiological relevance of this condition is emphasized by the results of the post-rest protocol, where we observe that the calcium transient increases for increasing rest times, even when SR Ca load is declining (see Figure S6 in Appendix S1). These simulations also agree with the experimental results by Picht et al [9], linking calcium alternans without fluctuation in SR Ca load with post-rest potentiation. Together, this suggests that the mechanism underlying alternans termed “R” in our simulations can explain the experimental findings of Picht et al. Alternatively, cytosolic calcium alternans at constant diastolic values of SR calcium loading has been explained by Rovetti et al [24] as a combination of effects involving RyR2 recovery, recruitment and randomness of the calcium release units (CaRUs). Their model produces calcium transients that are desynchronized in different parts of the cells, which is in accordance with results from calcium overloaded rat ventricular myocytes by Diaz et al [23]. However, it has been recently shown in human atrial myocytes with normal SR calcium load that calcium release is typically synchronized during pacing-induced calcium alternans [11], [25].

In concordance with recent experiments [11], we also show that although oscillations in SR Ca load are present, they are not always responsible for calcium alternans. In our analysis of the model, when the SR is loaded above a certain threshold all RyR2s are activated by *c_SR_*, since all luminal calcium-binding sites in the RyR2 are filled. Oscillations in *c_SR_* can therefore not drive calcium alternans. By contrast, oscillations in RyR2 refractoriness are still able to maintain calcium alternans. Inactivation is dependent on the calcium concentration at the dyadic space, so that a larger calcium depletion produces a bigger fraction of inactivated RyR2 channels, which in turn may cause incomplete RyR2 recovery at fast pacing rates. Under such conditions, there is a steep relation between the calcium released from the SR and the fraction of the recovered RyR2s [26]. This situation is favored when both RyR2 activation and recovery from inactivation are slowed.

We have shown that is indeed the case considering a situation where both the SR calcium and subsarcolemma calcium concentration remain fixed (see Section 2 in Appendix S1). Under this condition the concentration of calcium in the dyadic space increases when the L-type calcium channels open (due to voltage) triggering the release of calcium from the SR. Therefore, the presence of alternans can only be explained because of nonlinearities in the release resulting from the dynamics of the RyR2. A full analysis of this nonlinearity, shows that RyR2 dynamics can indeed lead to calcium alternans (Figures S4 and S5 in Appendix S1).

### Physiological and Pathophysiological Predictions of the Model

A number of studies have reported on associations between cardiac rhythm disturbances and abnormal SR function. This includes changes in the phosphorylation state of phospholamban and the RyR2 that are known to modulate SR calcium loading [27] and RyR2 opening [25], [28], [29], respectively. These studies include reports linking heart failure [30], CPVT [31] and atrial fibrillation to increased phosphorylation and/or calcium release through the ryanodine receptor [25], [28], [29], [32]. An increase in RyR2 opening could result from longer and/or more frequent RyR2 opening. Longer opening in turn may result from slower RyR2 inactivation and/or faster RyR2 recovery from inactivation, while more frequent opening would require faster RyR2 activation and recovery from inactivation. Our analysis of the model shows that faster RyR2 activation as well as faster RyR2 recovery prevents the induction of calcium alternans and shifts the threshold for its induction towards higher stimulation frequencies, making them unlikely to be mechanisms underlying the induction of calcium alternans. In accordance with this prediction of our analysis, it has recently been shown that drugs that increase the frequency of RyR2 openings but decrease the open time of individual events have antiarrhythmic effects [33], [34].

By contrast, our results show that a slowing of RyR2 inactivation would promote calcium alternans and lower the beating frequency where calcium alternans is induced suggesting that these arrhythmias are likely associated with a slowing of RyR2 inactivation. In accordance with this notion, slowing of the termination of RyR2 calcium release has been reported in patients prone to arrhythmia [35]. Consequently, pharmacological interventions that decrease RyR2 opening by increasing RyR2 inactivation are expected to be antiarrhythmic by preventing both spontaneous SR calcium release and the induction of calcium alternans. Our analysis of the model also predicts that antiarrhythmic candidates such as tetracaine that prevent RyR2 opening by slowing RyR2 activation could be proarrhythmic because they favor the induction of calcium alternans. In line with this, it has been shown experimentally that tetracaine favors the induction of non-uniform beat-to-beat responses at lower stimulation frequencies in human atrial myocytes [25], [36].

Another field gaining increasing attention is genetic mutations linked to abnormal RyR2 function or SR calcium loading [20], [21]. Here, current paradigms are that mutations causing SR overload or calcium leak are likely to be arrhythmogenic by promoting calcium release-induced afterdepolarizations [37]. However, as pointed out above, the present model predicts that mutations that increase RyR2 open probability by increasing RyR2 activation may be antiarrhythmic because they are expected to prevent the induction of calcium alternans.

On the other hand, our results also suggest that mutations which decrease RyR2 open probability by reducing RyR2 activation are likely arrhytmogenic because they induce calcium alternans at lower beating rates. In accordance with this prediction, the first mutation in the RyR2 associated with ventricular fibrillation (A4860G), which dramatically reduces RyR2 opening, was recently described and shown to be associated with a strong reduction in luminal calcium activation of the RyR2 [38]. Together, this shows that the present model may be useful to understand and predict the relationship between molecular alterations that affect RyR2 refractoriness and rate-dependent beat-to-beat changes in the intracellular calcium transient in isolated cardiomyocytes.

### Conclusion

The present study has used a well characterized rabbit numerical ventricular myocyte model and a dynamic clamping protocol to systematically investigate how fundamental RyR2 properties such as activation, inactivation, and recovery from inactivation as well as SR calcium loading contribute to determine the frequency dependent induction of cytosolic calcium alternans. This approach allows a mapping of the beat-to-beat response as a function of RyR2 activation and inactivation as well as the identification of domains where SR calcium load and/or RyR2 recovery from inactivation contribute to the induction of calcium alternans. It also allows the identification of transition zones where one predominant mechanism is substituted by another, and a characterization of how the transition zones depend on the stimulation frequency or the RyR2 recovery time. Importantly, the developed clamping protocols can also be used to study the mechanism behind alternans in other cardiac myocyte models.

A consequence of our study relevant to the analysis of other cardiac cell types is that even when experimental data shows concurrent alternations in calcium load and the cytosolic calcium transient, this does not necessarily imply that alternation in calcium load is the underlying mechanism.

## Supporting Information

Appendix S1 **Supplemental material with further information on the modifications of the RyR2 properties in the model by Shannon et al. necessary to obtain cytosolic calcium alternans.** It also includes some extra simulations, using an action potential clamp to eliminate potential interference from alternations in action potential amplitude or duration. Finally, it provides a mathematical study of the instability leading to calcium alternans and a more detailed analysis of the post-rest potentiation of the calcium transient. The appendix includes the following sections and figures: 1. – Parameters for the dynamics of RyR2 (with Figures S1, S2 and S3). 2.- Return map analysis of calcium alternans at constant SR load (with Figures S4 and S5). 3. – Restitution of calcium release (with Figure S6).
(PDF)
