# Supplementary material for: Dependency of Calcium Alternans on Ryanodine Receptor Refractoriness
Source: PLoS One. 2013 Feb 4;8(2):e55042. doi: 10.1371/journal.pone.0055042 (PMC3563653; doi:10.1371/journal.pone.0055042)
Supplement: Appendix S1 — Supplemental material with further information on the modifications of the RyR2 properties in the model by Shannon et al. necessary to obtain cytosolic calcium alternans. It also includes some extra simulations, using an action potential clamp to eliminate potential interference from alternations in action potential amplitude or duration. Finally, it provides a mathematical study of the instability leading to calcium alternans and a more detailed analysis of the post-rest potentiation of the calcium transient. The appendix includes the following sections and figures: 1. – Parameters for the dynamics of RyR2 (with Figures S1, S2 and S3). 2.- Return map analysis of calcium alternans at constant SR load (with Figures S4 and S5). 3. – Restitution of calcium release (with Figure S6). (PDF) [file pone.0055042.s001.pdf]

SUPPLEMENTAL MATERIAL

DETAILED METHODS

In this supplemental material we provide information on the modifications of the RyR2 properties in the model by Shannon et al. [1] necessary to obtain cytosolic calcium alternans. Besides, we repeat some of the simulations in the main manuscript, using an action potential clamp to eliminate potential interference from alternations in action potential amplitude or duration. We also provide a mathematical study of the instability leading to calcium alternans and a more detailed analysis of the post-rest potentiation of the calcium transient.

1. – Parameters for the dynamics of RyR2

1.1 Dynamics and nomenclature

Intracellular calcium dynamics has been modeled using the Shannon et al. [1] model for rabbit ventricular myocytes, as present in the repository CellML [2]. In this model the cell is divided into four different compartments where calcium can be tracked. Namely, junctional and subsarcolemma areas, close to the cell membrane, the cytosol and the SR space. Calcium concentrations in each compartment are labeled c_{jct}, c_s, c_{cyt}, and c_{SR}, respectively. The RyR2 gating dynamics follows the formulation developed by Stern et al. [3] (see Figure S1). The system of equations for the RyR2 reads:

$$
\begin{aligned}
\frac{dR}{dt} &= k_{im}RI - \tilde{k}_i c_{jct}R - \tilde{k}_a c_{jct}^2 R + k_{om}O \\
\frac{dO}{dt} &= \tilde{k}_a c_{jct}^2 R - k_{om}O - \tilde{k}_i c_{jct}O + k_{im}I \\
\frac{dI}{dt} &= \tilde{k}_i c_{jct}O - k_{im}I - k_{om}I + \tilde{k}_a c_{jct}^2 RI \\
\frac{dRI}{dt} &= k_{om}I - \tilde{k}_a c_{jct}^2 RI - k_{im}RI + \tilde{k}_i c_{jct}R
\end{aligned}
\qquad (1)
$$

with $\tilde{k}_a = k_a/k_{CSR}$, $\tilde{k}_i = k_i k_{CSR}$, being k_a and k_i constants and k_{CSR} a function of the SR calcium load which incorporates implicitly the dependence of activation and inactivation on calsequestrin

$$
k_{CSR} = Max_{SR} - \frac{Max_{SR} - Min_{SR}}{1 + \left(\frac{EC_{50-SR}}{c_{SR}}\right)^{H_{SR}}} \qquad (2)
$$

The flux of calcium released from the SR reads:

$$
J_{SRCaRel} = k_s O\,(c_{SR} - c_{jct}) \qquad (3)
$$

The parameters specifying RyR2 properties in the rabbit model by Shannon et al. are modified in order to investigate the effects of RyR2 activation, inactivation and recovery from inactivation rates on the intracellular calcium dynamics. Activation and inactivation rates were changed systematically to test their effects on the dynamics. This is implemented

changing the constants k_a and k_i defined above. Regarding the recovery time of RyR2, three different values were used to characterize the effects of recovery from inactivation on the appearance of alternans.

Table S1 shows the original parameters in [1], and the corresponding changes used in our simulations. Using this nomenclature, activation, inactivation and recovery from inactivation correspond to parameters k_a, k_i, and k_{im} respectively. In our analysis k_a takes values in the interval 0.1-10.0 $mM^{-2}ms^{-1}$, and k_i takes values in the interval 0.005-1.0 $mM^{-1}ms^{-1}$. For the original Shannon model, k_a=10 $mM^{-2}ms^{-1}$ and k_i = 0.5 $mM^{-1}ms^{-1}$ respectively. On the other hand, k_{im} corresponds to the inverse of the recovery time from inactivation τ_r (k_{im}=1/τ_r). Values of τ_r=200 ms, 750 ms and 1500 ms are used in simulations. However, the benchmark value used in most of the simulations is 750 ms, while the recovery time from inactivation is 200 ms in [1].

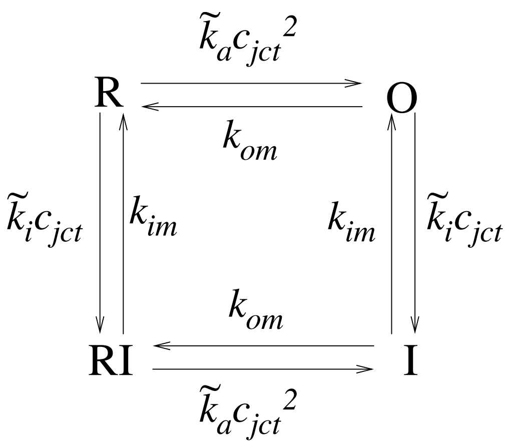

Figure S1: Schematic representation of the RyR2 gating dynamics. Four state Markov model, with O being the open state, R the recovery state, and RI and I two inactivated states. The dynamics of activation (opening), inactivation and recovery of the RyR2 are given by the rates $\tilde{k}_a = k_a/k_{CSR}$, $\tilde{k}_i = k_i k_{CSR}$, and k_{im}, where k_{CSR} is a non-dimensional function of the calcium concentration on the SR, as seen in Eq. (2). We also define a recovery time as the inverse of the recovery rate $\tau_r = 1/k_{im}$.

1.2 Activation and inactivation rates. Calcium alternans.

Isolated rabbit cardiomyocytes often exhibit long transient alternans at high frequency pacing (5Hz) [4]. The model by Shannon et al. with the original parameters, however, fails to present alternans at these high frequencies (Figure S2A). This is largely independent of the recovery time of the RyR2. See, for instance, Figure S2B, which shows a regular calcium response even when the recovery time is changed from 200 ms to 750 ms. On the contrary, a reduction of activation and inactivation rates to, respectively, 75% and 50% of the original values produces long transient calcium alternans at 5Hz (See Figure S2C). As indicated in the manuscript, a further lowering of activation and inactivation rates results in persistent alternans even at normal pacing rates (~3Hz). We conclude that changes of activation and/or inactivation of around 50% in the original parameters in [1] reproduce the general behaviour of many healthy cardiomyocytes. Further reductions in activation or inactivation would correspond to pathological conditions as discussed in the last section of the manuscript.

A)

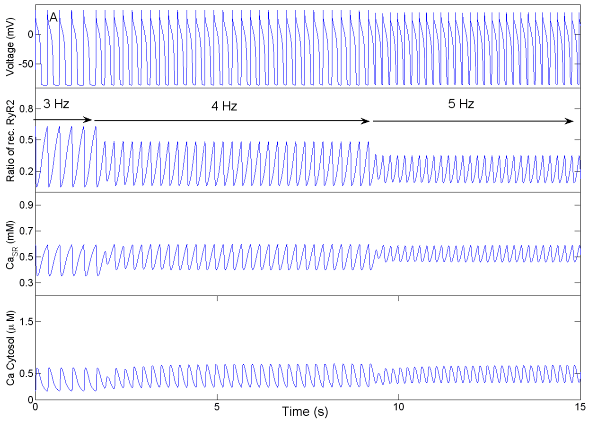

B)

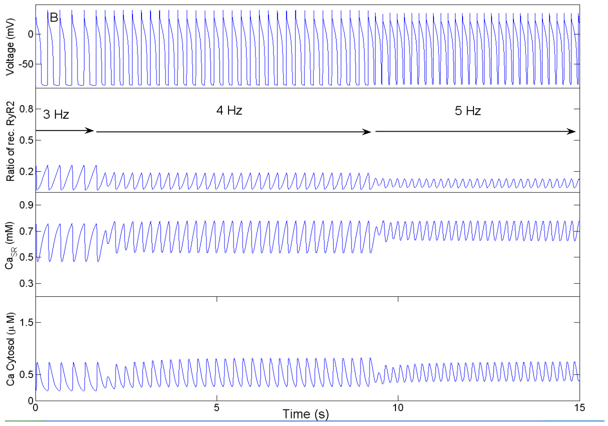

C)

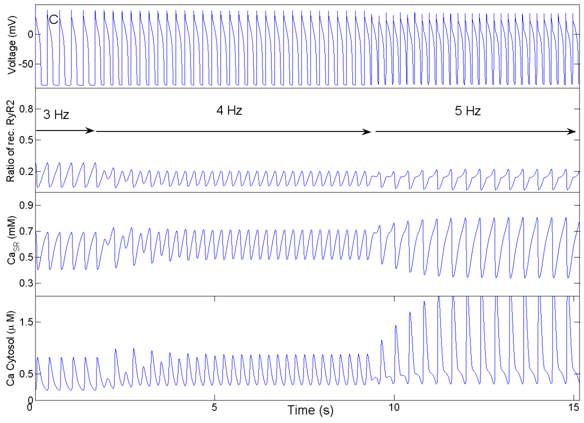

Figure S2: Voltage and calcium dynamics at fast pacing rates. In each panel we show, from top to bottom, the transmembrane voltage, level of recovered RyR2s, SR Ca content, and time evolution of cytosolic calcium, as pacing frequency is increase from 3Hz, to 4Hz, and finally 5Hz. Parameters of the model are changed in each panel. A) Original parameters in Shannon et al. [1] for activation, inactivation and recovery from inactivation of the RyR2. B) Recovery from inactivation time scale increased to 750 ms (instead of the original 200 ms). C) RyR2 activation and inactivation reduced to, respectively, 75% and 50% of their original values.

1.3.- Action Potential clamp

In order to check that the alternans we see in the numerical simulations are due to a calcium, and not to a voltage, instability, we repeated the simulations leading to Figure 3D, using action potential (AP) clamp. We show the results in Fig S3, where we compare the cases without (A) and with (B) AP clamp. The AP clamp is implemented using the same AP waveform as in [5], given by

$$V(t) = \begin{cases} V_0 + \Delta V \sqrt{1 - \left(\dfrac{t - mT}{APD}\right)^2} & mT \le t \le mT + APD \\ V_0 & mT + APD < t < (m+1)T \end{cases} \tag{4}$$

where V_0 is the resting potential, that we take V_0=-85.8 mV, ΔV=V_{max}-V_0=125 mV and APD is the action potential duration, whose value changes depending on the frequency of stimulation (at 3Hz APD=168 ms). We have checked that for a fixed frequency rate these parameters are fairly independent on the activation and inactivation rates.

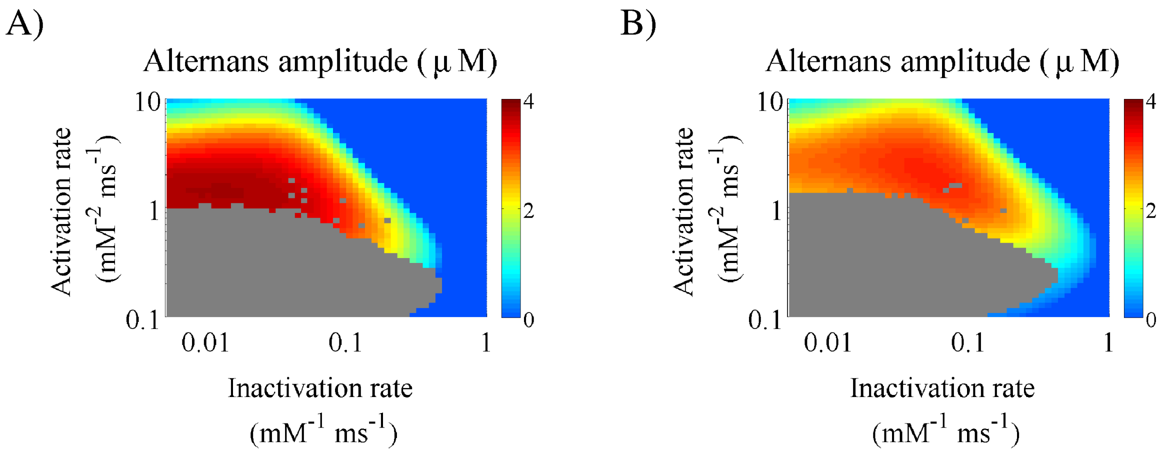

Figure S3: Effects of AP clamp on the appearance of calcium alternans. Color-code graphs of the amplitude of alternations in the calcium transient amplitude as a function of RyR2 activation and inactivation at a pacing rate of 3Hz, without (A) and with (B) AP clamp.

From Figure S3 it is clear that the borders delimiting the transition to alternans do not change appreciably. There is just a change in the amplitude of calcium alternans, which becomes slightly smaller with the AP clamp due to the elimination of the feedback with the voltage dynamics.

2.- Return map analysis of calcium alternans at constant SR load

In this section we analyse how the dynamics of the RyR2 can lead to alternans. We show that a slow recovery from inactivation, together with low activation and non-negligible inactivation can lead to an instability in the RyR2 dynamics. For this study we construct and analyse a return map relating the level of recovered RyR2s at two consecutive beats.

Return maps have provided a very useful tool to study the onset of alternans. They were first used to study voltage alternans, where it was observed that there exists a relation between the APD at beat n and the previous diastolic interval (DI, time elapsed between the end of an action potential and the beginning of the following one), so one can write the map $APD^{n}=f(DI^{n-1})$. Assuming a constant stimulation period $T=APD+DI$, this map becomes $APD^{n}=f(T-APD^{n-1})$ that, by means of standard bifurcation analysis, can be shown to present a period-doubling bifurcation when the slope of the map f is larger than 1. In the ensuing instability, the APD alternates from beat to beat, resulting in voltage alternans. This analysis was extended to the case of calcium alternans due to a steep relation between calcium load and calcium release by Shiferaw et al. [5], where they constructed a map relating SR calcium at two consecutive beats $c_{SR}^{n+1}=g(c_{SR}^{n})$. They showed that, indeed, the dynamics presented alternans when the slope of the map fulfilled the condition $dg/dc_j<-1$, resulting in a period-doubling instability.

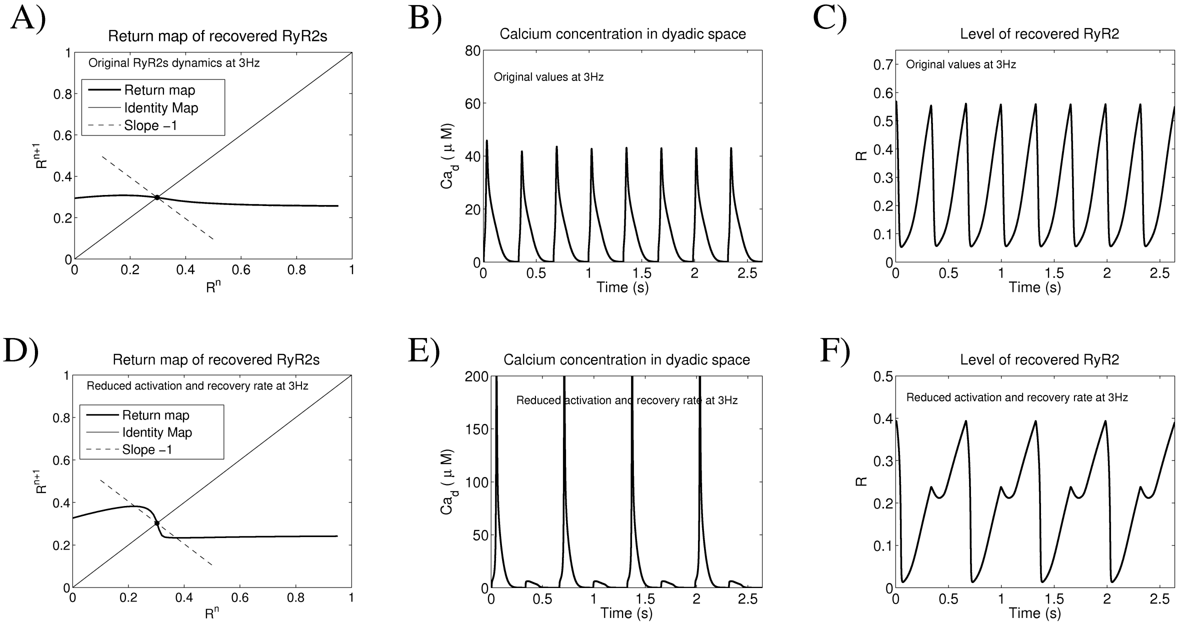

Figure S4: Return map analysis. Return maps, dyadic calcium dynamics and level of recovered RyR2s at 3Hz for (A-C) RyR2 parameters in the original Shannon model (SR load at 0.55 mM) and (D-F) RyR2 parameters k_a=1.4 mM^{-2}ms^{-1}, $k_i = 0.5$ mM^{-1}ms^{-1}, τ_r=750 ms (SR load at 1.2 mM).

We have used the same approach to study the origin of calcium alternans when the underlying mechanism is that of slow recovery from inactivation. For that, we fix both the SR calcium load, and the calcium concentration in the subsarcolemma, and stimulate the cell with the AP clamp given in Eq. (4) to fix the opening voltage-dependent gates of I_{CaL} at each beat. We then construct a map relating the level of recovered RyR2s (state R in Figure S1) at two consecutive beats $R^{n+1}=h(R^{n})$. Since the rest of variables are fixed, the appearance of alternans must be necessarily linked to an instability in the RyR2 dynamics, which is reflected as a bifurcation in this map.

In Figure S4 we show the return map $R^{n+1}=h(R^{n})$ and the corresponding dyadic calcium concentration and level of recovered RyR2s, for parameters that give rise to stable calcium cycling (Figure S4A-C) and calcium alternans (Figure S4D-F). The steady state value is given by $R^*=h(R^*)$, and the instability condition $\mathrm{d}h(R^*)/\mathrm{d}R<-1$. As expected, the slope of the return map is shallow in the first case, but steep in the second. Then, the basic dynamics of the RyR2 is the following: after a beat with large release, the RyR2 inactivates strongly and, because of the slow recovery, the level of recovered RyR2s at the next stimulation does not increase enough to trigger a large release, leading to a small calcium transient. This produces a very slight inactivation, so the RyR2 is recovered in the following stimulation, resulting again in a large release.

These results are largely independent on the value we choose for the concentration of calcium in the subsarcolemma (we fix it at an equilibrium level of 0.1 μM). They depend strongly, however, on the ratio between the recovery time and the pacing period, on the presence of low activation and on the specific value of the SR load. Regarding the SR load, it must be large enough to trigger a response when activated by I_{CaL}. For large activation, alternans does not appear since the release turns out to be largely independent of the level of recovered RyR2. In this situation the return map becomes shallow. At low activation, on the other hand, a given value of SR load results in a large release only when the RyR2 is sufficiently recovered. This dependence of release on the level of recovered RyR2s results in a steep slope of the return map, separating two regions with different dynamics. In Figure S4D-F we have chosen a value of inactivation that gives alternans due to the R mechanism alone in the full model, and we have taken the corresponding load from the value in the full non-clamped evolution.

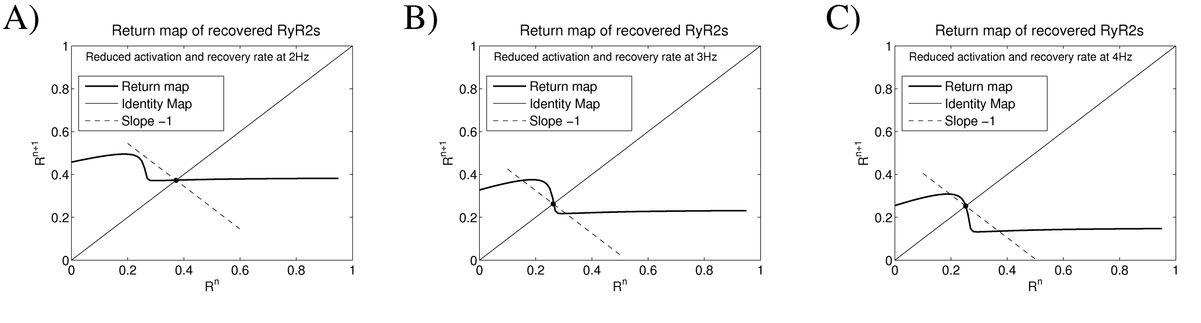

Figure S5: Return maps and cytosolic calcium dynamics as a function of stimulation frequency. A) f=2Hz, B) f=3Hz, and C) f=4Hz. RyR2 parameters k_a=1.4 $\mathrm{mM^{-2}ms^{-1}}$, $k_i = 0.5$ $\mathrm{mM^{-1}ms^{-1}}$, τ_r=750 ms with SR load at 1.2mM.

The other relevant feature is that the recovery time from inactivation cannot be of the same order of the pacing period. Given a recovery time τ_r, alternation in the dynamics only appears for pacing periods significantly shorter than τ_r, as can be observed in Figure S5. There, as we decrease the period of stimulation (increase the frequency), the steady state moves from the shallow part of the curve (Figure S5A) to the steep part (Figure S5B-C), corresponding to the passing from normal calcium cycling to alternans.

3. – Restitution of calcium release

There are different aspects of the results regarding calcium release restitution that we would like to comment further. First, activation and inactivation kinetics determine whether the SR Ca content before each depolarization is higher or lower than the equilibrium concentration set by the Na/Ca exchanger and the SR calcium pump when the cardiomyocyte is at rest. This feature is important in order to properly reproduce the post-rest potentiation in the calcium transient, as observed in recent experiments [6]. The reason is that without changes in activation and inactivation rates, such as those introduced in the manuscript, the SR Ca load typically increases with the resting time, which is in clear contrast with these experiments. Second, in [6], Pitch et al. showed that calcium released from the SR increased after two-three seconds without pacing, even though both the I_{CaL} amplitude and the SR Ca loading decreased slightly. We find that it is not sufficient to modify RyR2 activation and/or inactivation rates (with respect to the original values in [1]) to accommodate this result. A longer time for the recovery of the RyR2 from inactivation is also required.

One must notice that the experiments by Picht et al. were done at room temperature, while the Shannon model was validated at physiological temperature. Indeed, the model does have temperature corrections for some, but not all, the currents. Of particular relevance to us, neither the SR release, nor the RyR2 dynamics present any temperature dependence. Because the results obtained changing the temperature in the model could be misleading, we have performed simulations at physiological temperature, looking only for a qualitative agreement with the experiments.

We took into account that there are three different aspects of calcium handling that may lead to post-rest potentiation: 1) An increase in the SR calcium load, 2) an increase in the level of RyR2s recovered or 3) a time dependent increase in the I_{CaL} amplitude. To study these effects we measured the calcium released from the SR using a S1-S2 protocol (Figure S6A). We stimulated the cell at a constant interval S1 of 1Hz until it reached steady state. Then, we applied a S2 stimulus at increasingly longer intervals.

Figure S6B shows the SR Ca concentration transient after reaching steady state at a pacing period of 1 Hz, compared with the fractional release divided by the peak in I_{CaL}. While in the experiments the SR Ca concentration at rest is lower than the one reached at 1Hz pacing (resulting in a decreasing trend with time), in the model by Shannon et al., the opposite behavior is observed. With the above-mentioned changes in activation and inactivation in the dynamics of the RyR2 (a decrease in activation and inactivation rates) the correct trend is, however, recovered. The bottom panel in Figure S6B computes the fractional release as a function of the S2 interval, showing that it increases, resulting in post-rest potentiation. This

clearly cannot be due to an increase in SR Ca concentration, neither to a slow recovery of I_{CaL}, since it occurs on a much faster time scale (see Figure S6B top and S6C). In fact, it corresponds to the recovery time from inactivation of the RyR2, which regulates the refractoriness of calcium release.

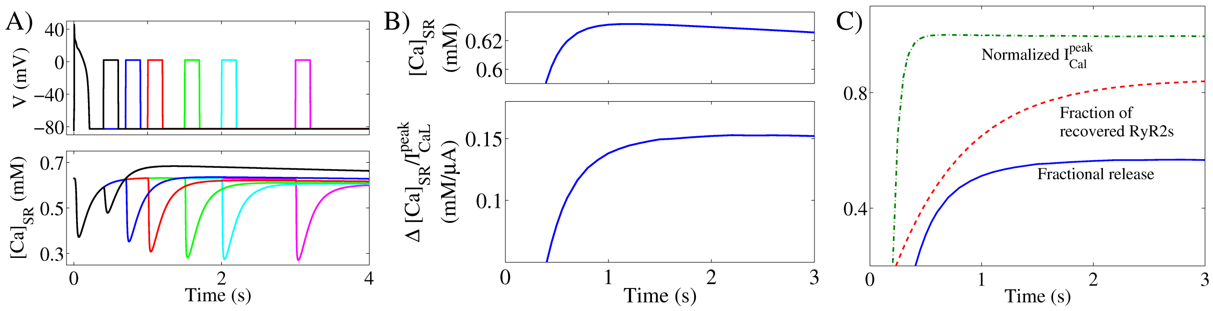

Figure S6: S1S2 protocol showing post-rest potentiation of SR calcium release. The cell is stimulated with an external current at a constant S1 interval of 1s, until it reaches steady state. Then, a S2 stimulus is given at different intervals, under voltage clamp. In panel A) we show the resulting voltage and the SR calcium release transient for several selected S2 intervals. B) Corresponding SR Ca concentration prior to the S2 stimulus (top) and calcium gain (bottom). C) Blue solid line: Fractional release (defined as the decrease in SR Ca content with respect to the value before the stimulus). Dashed red line: Fraction of recovered RyR2. Dot-dashed green line: Peak I_{CaL} current after each S2 stimulus normalized with respect to the maximum peak current for any interval.

REFERENCES

1. Shannon TR, Wang F, Puglisi J, Weber C, Bers DM (2004) A mathematical treatment of integrated Ca dynamics within the ventricular myocyte. Biophysical Journal 87: 3351-3371.
2. Lloyd CM, Halstead MD, Nielsen PF (2004) CellML: its future, present and past. Prog Biophys Mol Biol 85: 433-450.
3. Stern MD, Song LS, Cheng H, Sham JS, Yang HT, Boheler KR, Ríos E (1999) Local control models of cardiac excitation-contraction coupling: A possible role for allosteric interactions between ryanodine receptors. J Gen Physiol 113:469-489.
4. Chudin E, Goldhaber J, Garfinkel A, Weiss J, Kogan B (1999) Intracellular Ca(2+) dynamics and the stability of ventricular tachycardia. Biophysical Journal 77: 2930-2941.
5. Shiferaw Y, Watanabe MA, Garfinkel A, Weiss JN, Karma A (2003) Model of Intracellular Calcium Cycling in Ventricular Myocytes. Biophysical Journal 85: 3666–3686.
6. Picht E, DeSantiago J, Blatter L, Bers D (2006) Cardiac alternans do not rely on diastolic sarcoplasmic reticulum calcium content fluctuations. Circulation Research 99: 740-748.

Table S1: Parameters used in the simulations.

Parameter	Shannon et al. value	This manuscript	Units
k_s	25	25	ms^{-1}
k_a	10	0.1-10	$mM^{-2}ms^{-1}$
k_{om}	0.06	0.06	ms^{-1}
k_i	0.5	0.005-1	$mM^{-1}ms^{-1}$
k_{im}	0.005=1/200	1/200, 1/750, 1/1500	ms^{-1}
EC_{50-SR}	0.45	0.45	mM
Max_{SR}	15	15	
Min_{SR}	1	1	
